# Thermal and Structural Behavior of Dioctadecyldimethylammonium Bromide Dispersions Studied by Differential Scanning Calorimetry and X-Ray Scattering

**DOI:** 10.1371/journal.pone.0044702

**Published:** 2012-09-06

**Authors:** Eloi Feitosa, Renata D. Adati, Per Hansson, Martin Malmsten

**Affiliations:** 1 Department of Pharmacy, Uppsala University, Uppsala, Sweden; 2 Department of Physics, São Paulo State University, Sao Jose do Rio Preto, Brazil; German Cancer Research Center, Germany

## Abstract

Dioctadecyldimethylammonium bromide (DODAB) is a double chain cationic lipid, which assembles as bilayer structures in aqueous solution. The precise structures formed depend on, *e.g.*, lipid concentration and temperature. We here combine differential scanning calorimetry (DSC) and X-ray scattering (SAXS and WAXS) to investigate the thermal and structural behavior of up to 120 mM DODAB in water within the temperature range 1–70°C. Below 1 mM, this system is dominated by unilamellar vesicles (ULVs). Between 1 and 65 mM, ULVs and multilamellar structures (MLSs) co-exist, while above 65 mM, the MLSs are the preferred structure. Depending on temperature, DSC and X-ray data show that the vesicles can be either in the subgel (SG), gel, or liquid crystalline (LC) state, while the MLSs (with lattice distance *d*  = 36.7 Å) consist of interdigitated lamellae in the SG state, and ULVs in the LC state (no Bragg peak). Critical temperatures related to the thermal transitions of these bilayer structures obtained in the heating and cooling modes are reported, together with the corresponding transition enthalpies.

## Introduction

In dilute aqueous solution, and above its gel-to-liquid crystalline temperature (T_m_≈45°C), dioctadecyldimethylammonium bromide (DODAB) self-assembles as unilamellar vesicles (ULVs) [1]–[3], providing potential application, *e*.*g*., as a non-viral vector in gene therapy [4]. DODAB concentration and solvent composition, as well as mechanical constraints (*e.g.*, sonication or extrusion), affect the vesicle structure [5]–[7]. Together, these and other factors allow tuning of vesicle properties for specific purposes and applications.

A wide range of methods has been used to investigate DODAB and other vesicle systems. Among these, differential scanning calorimetry (DSC) and X-ray scattering (SAXS and WAXS) offer particular opportunities for studying thermal and structural aspects of these systems. The profile of the DSC thermograms and X-ray scattering curves can be sensitively monitored as function of various factors, including lipid concentration, ionic strength, pH, equilibration time, or method of preparation, to reveal the structural organization of the lipid bilayers [5], [6], [8].

DODAB aqueous dispersions are usually prepared by the heating method at a temperature safely above its T_m_, typically 55–65°C [1], [9]. On cooling, the bilayer structures are preserved even though the aggregate structure can change [10], [11]. The dominating structures, however, are highly dependent on DODAB concentration [12]–[14]. The precise concentration range of single ULV or multilamellar structure (MLS) formation is still a subject of controversy [11]–[13].

Given the remaining uncertainties of the structures formed in this system, we here report on systematic combined investigations of structural and thermal properties in this system over a wide range of concentration and temperature, including systematic variations in sample history, to carefully map the structural and thermal transition in DODAB dispersions. In doing so, we demonstrate that ULV, ULV + MLS, and MLS are the dominating structures up to 1 mM, between 1 and 65 mM, and above 65 mM DODAB, respectively. Enthalpies and hysteresis are furthermore reported for the transition between these states at different DODAB concentrations.

**Figure 1:**
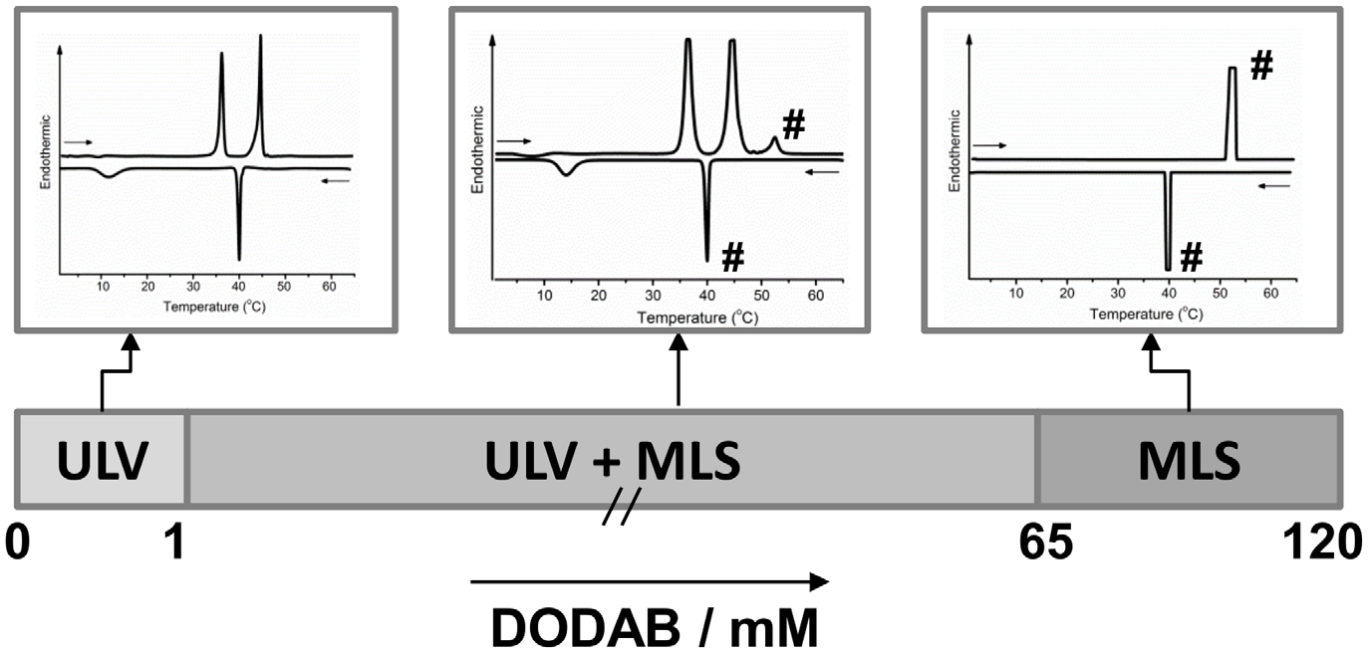
Bilayer structures and representative DSC thermograms in DODAB dispersions. Dominating DODAB bilayer structures in water at 1°C and representative heating/cooling thermograms for each structure at 1, 5, and 80 mM. On the thermograms, “#” identifies the endotherm and exotherm related to the MLSs. The remaining peaks are related to the ULVs. The horizontal arrows indicate heating or cooling scans.

## Materials and Methods

DODAB (>98%, Lot # 0001406711) was supplied by Sigma-Aldrich (St. Louis, USA), and used without further purification. Milli-Q quality water was used throughout for sample preparations.

### Preparation of DODAB Dispersions

DODAB dispersions were prepared by weighing the appropriate amount of DODAB, followed by water addition to obtain the desired concentration. DODAB/water mixtures were gently stirred magnetically during a few minutes at 65°C to obtain optically homogeneous dispersions, which were then cooled to room temperature (25°C) and equilibrated for the desired time prior to DSC or X-ray experiments.

**Figure 2:**
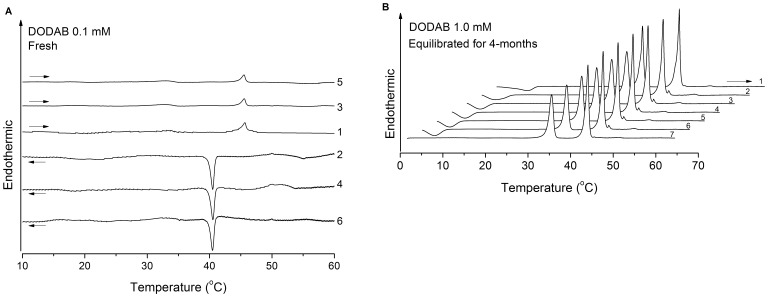
Thermograms of fresh and equilibrated DODAB dispersions. Sequence of (A) heating (curves 1, 3 and 5) and cooling (curves 2, 4 and 6) traces for fresh DODAB dispersion at 0.1 mM, obtained using null pre-scan time at 1 or 65°C. **(**B) Sequence of heating traces of four-month equilibrated DODAB dispersion at 1.0 mM obtained using null pre-scan time (exception for curve 7, 16 h). The offset between successive traces equals 3.6°C. The horizontal arrows indicate heating or cooling scans.

### DSC Experiments

A VP-DSC Microcalorimeter (Microcal, Inc., Northampton, USA) was used to collect DSC data, and the Origin® 7.0 software (supplied by the manufacturer) used to record and analyze the data. Details on the equipment setup can be found elsewhere [12]. Briefly, degassed sample and reference (water) were poured into the (Tantalum, 0.5 ml, non-capillary type) twin cells, and experiments performed by heating or cooling the sample at the desired scan rate (20°C/h, if not stated otherwise) in the temperature range 1–65°C. Thermograms were recorded as changes in heat capacity, at constant pressure, as a function of temperature.

**Figure 3:**
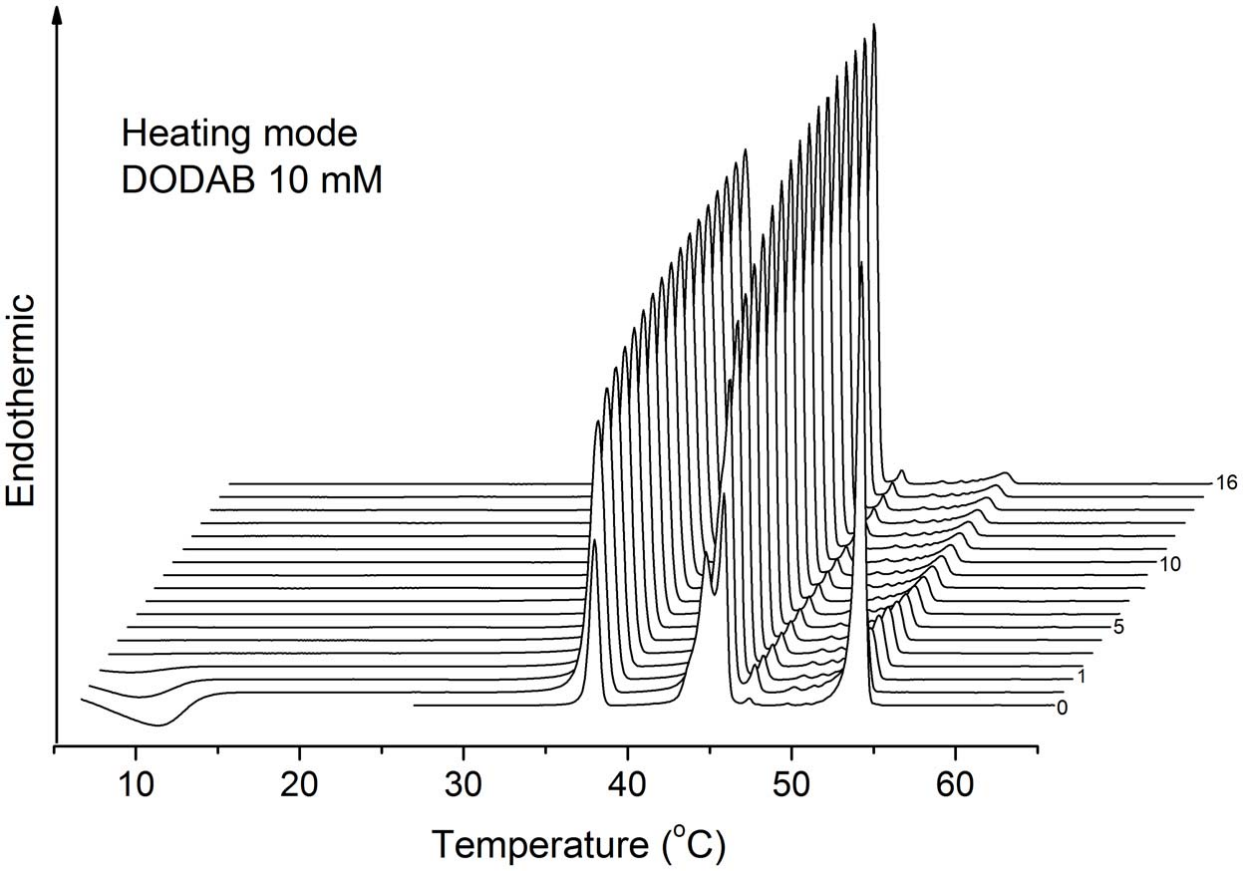
Heating thermograms of a 10 mM DODAB dispersion. Sequence of selected heating thermograms for DODAB 10 mM in water, obtained for T_i_  = 25°C using null pre-scan time, or T_i_  = 1°C using pre-scan times 0, 1, 2, 3,…, 16 h, as indicated. Offset between two successive traces is 0.6°C. The horizontal arrow indicates heating scan.

**Figure 4:**
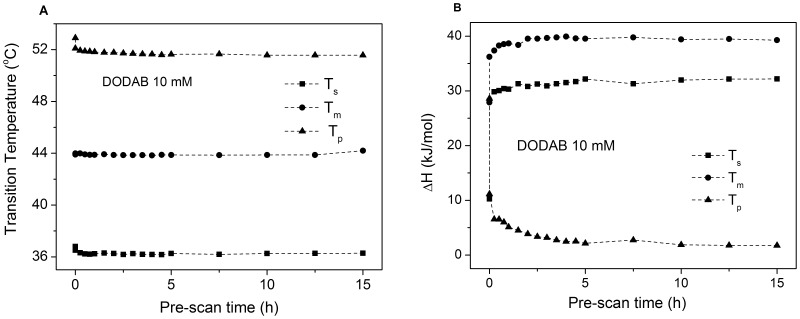
Efect of equilibration time on critical temperatures and enthalpy of DODAB dispersions. Effects of pre-scan time on T_s_, T_m_, and T_p_ (A) and transition enthalpies (B) for DODAB 10 mM in water, obtained from the thermograms data analysis.

In line with the conventional nomenclature in the area, subgel, gel, and liquid crystalline states refer to the ordered (“freezed”), semi-ordered (“tilty”), and disordered (“melted”) conformational state of the lipid chains in the bilayer structures, respectively. The critical temperatures T_s_ (T’_s_) and T_m_ (T’_m_) account for the gel-subgel and gel-liquid crystalline transition temperatures, respectively of the lipids in the vesicle bilayers in the heating (cooling) process. Similarly, T_p_ (T’_p_) refer to the subgel-liquid crystalline transition temperature of the lipids in the multilamellar structures (MLS) in the heating (cooling) process. Coagel, finally refer to MLS below T_p_.

### X-ray Scattering (SAXS and WAXS)

Simultaneous SAXS and WAXS measurements on DODAB/water samples were performed using a MBraun instrument (Graz, Austria). The dispersions were placed in 2.0 mm diameter Mark-tubes made of glass (Hilgenberg GmbH, Malsfeld, Germany), which were then flame-sealed. A Kratky camera with line collimation was used. CuKα radiation (λ = 1.542 Å) was generated using a Seifert Iso-Debyeflex 3003 generator operating at 2 kW, 50 kV and 40 mA. The camera uses a multiplex to record both small (SAXS) and wide (WAXS) angle scattering. The diffraction pattern was recorded by a MBraun linear position sensitive detector PSD 50 M and stored digitally. The exposure time was 1 h for each sample. According to Bragg's law, the scattering angle (2*θ*) is related to the scattering vector *q* by *q = 4π sinθ/λ*, and the lattice spacing *d = 2π/q_Bragg_*, where *q_Bragg_* is the *q* vector at the peak maximum [15]. The sample-detector distance was fixed at 300 nm.

**Figure 5:**
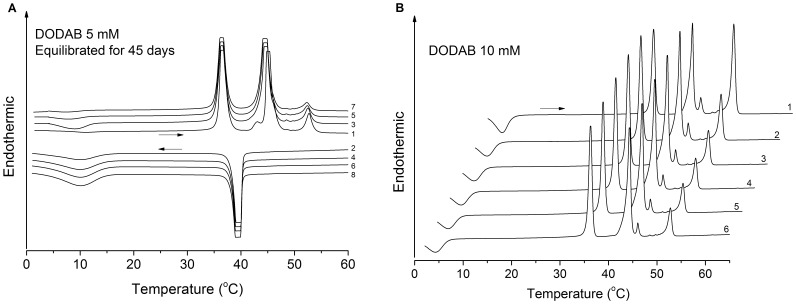
Representative thermograms of 5 and 10 mM DODAB dispersions. (A) Successive heating (curves 1, 3, 5, 7) and cooling (curves 2, 4, 6, 8) traces for a DODAB dispersion at 5 mM equilibrated for 45 days, using a pre-scan time of 15 min. (B) Sequence of heating traces (curves 1–6) for fresh DODAB dispersion at 10 mM obtained using a null pre-scan time. Offset between two successive traces is 2.5°C. The horizontal arrows indicate heating or cooling scans.

## Results and Discussion

### DODAB Bilayer Structures

Up to 120 mM in the range 1–70°C, DODAB dispersions display the following thermal transitions: an exotherm at T_e_≈5°C and three endotherms at T_s_≈36°C, T_m_≈45°C, and T_p_≈53°C, on heating, and two exotherms at T’_m_ = T’_p_≈40°C and T’_s_≈15°C, on cooling. As will be discussed in detail below, DSC and SAXS data demonstrate a progression from unilamellar vesicles (ULVs) at DODAB concentrations below 1 mM, to co-existence of ULVs and multilamellar structures (MLSs) in the range 1–65 mM, and finally, to MLSs up to 120 mM (Figure 1, Table S1).

Up to 1 mM, our DSC data suggest that DODAB dispersions are rich in ULVs, which display two endotherms at T_s_ and T_m_, characteristic of the subgel (SG)-to-gel and gel-to-liquid crystalline (LC) transitions, respectively, in line with previous reports in literature [1], [10], [11]. In the cooling mode, the reverse LC-to-gel and gel-to-SG exotherms are detected at T’_s_ and T’_m_, respectively [16].

Above 65 mM, there is a single SG-to-LC endotherm at T_p_≈53°C on heating and the reverse LC-to-SG exotherm at T’_p_≈40°C on cooling (notice that T’_p_ = T’_m_≈40°C). Above T_p_, the MLS revert to ULVs, demonstrated by the SAXS and WAXS curves (discussed below) displaying no Bragg peak.

Between 1 and 65 mM DODAB, the dispersions contain both ULVs and MLSs, the relative amount of MLSs increasing with DODAB concentration, and that of ULVs decreasing, to completely disappear around 65 mM. The thermal behavior of these co-existing structures is similar to those of the single ULV and MLS species.

### DODAB Thermal Transitions

The T_s_- and T_m_-endotherms, as well as their T’_s_ and T’_m_ reverses, are undoubtedly related to the ULVs [1], [16], while the T_p_-endotherm and its reverse T’_p_-exotherm are related to MLSs [11], [13], [16]. The physical meaning of the T_p_-endotherm has not, however, been conclusively elucidated so far. In a previous study, we reported on the T_p_-endotherm for DODAB at 5 mM, which was subsequently associated to the presence of dispersed lamellar structures [1], [12]. More recently, Coppola et al. [13] related it to a structural transition from ULVs + lamellar fragments-to-MLVs (multilamellar vesicles) that takes place above 10 mM DODAB, while Wu et al. [11] referred to it as the coagel-to-LC transition, occurring above 7.5 mM DODAB.

As will be discussed below, the DSC data indicate that the T_p_-transition accounts for the SG-to-LC transitions within the MLSs, whose reverse transition takes place at T’_p_ ≈ 40°C. The SAXS and WAXS data, in turn, indicate that below and above T_p_, the dominating structures are multilamellar and unilamellar, respectively. Together, these findings suggest that the T_p_-endotherm for the MLSs has similar physical meaning as the T_m_-endotherm for ULVs.

**Figure 6:**
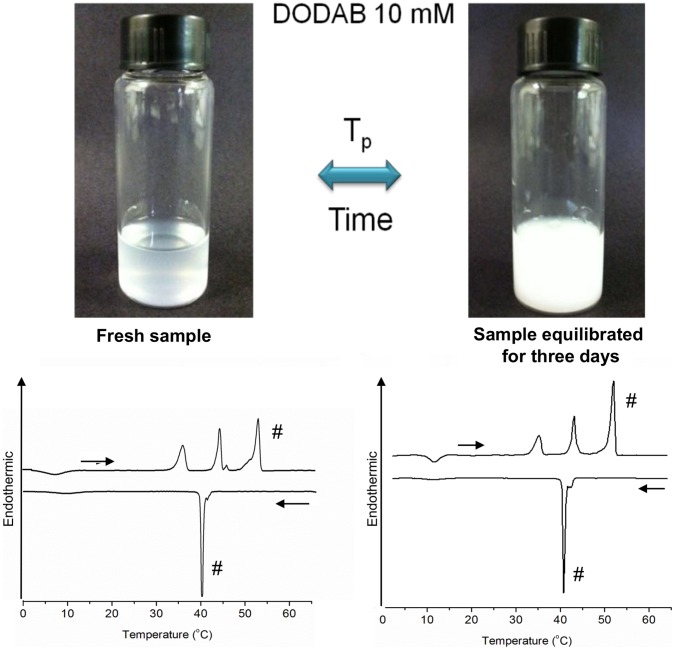
Pictures of a fresh and an equilibrated 10 mM DODAB dispersion and representative thermograms. Top: pictures taken at 25°C of fresh (left) and equilibrated for three days (right) DODAB dispersion at 10 mM. Bottom: Respective heating and cooling DSC thermograms showing that the relative intensity of the T_p_-endotherm is higher for the equilibrated than for the fresh sample. On the thermograms, “#” identifies the endotherm and exotherm related to the MLSs and the horizontal arrows indicate heating or cooling scans.

### Unilamellar Vesicles (ULVs)

Figure 2A depicts a series of heating and cooling thermograms for a fresh aqueous dispersion of DODAB at 0.1 mM. Similar traces were also obtained for the same dispersion equilibrated for six months at 25°C (not shown), indicating considerable vesicle stability. As shown, all thermograms present small single gel-LC transitions at T_m_ = 45.5°C and T’_m_ = 40.5°C in the heating and cooling modes, respectively, with a thermal hysteresis ΔT_m_ = 5°C, characteristic of ULVs [3], [16]. Since the T_p_-endotherm is not discernible, ULVs are the preferred structure at 0.1 mM.

At 1.0 mM, the heating thermograms for a 4-month equilibrated dispersion displays the T_s_- and T_m_-endotherms and their reverse T’_s_- and T’_m_-exotherms in the cooling mode (Figure 2B), characteristic of vesicles, with hysteresis ΔT_s_≈22°C and ΔT_m_ = 5°C, respectively [16]. Thus, the T_p_-endotherm, characteristic of the SG-to-LC transition of MLSs [10], [11], starts appearing at 1.0 mM DODAB, which was consequently taken as the onset of the MLS formation (Figure 1). The intensity of this transition does not change with the longer equilibration time of 16 h at 1°C (curve 7), indicating co-existence of ULVs/MLSs and that the dispersion is dominated by ULVs (larger T_m_- than T_p_-endotherm). Similar thermograms were obtained for fresh 1 mM DODAB dispersion (not shown).

**Figure 7:**
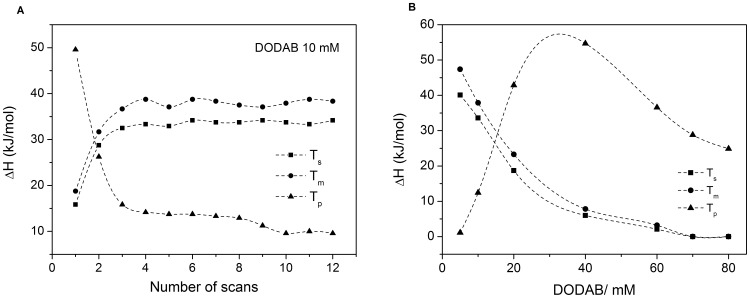
Effect of scan number and DODAB concentration on the transition enthalpies. (A) Effect of scan number on the T_s_-, T_m_-, and T_p_-enthalpies of DODAB dispersion at 10 mM, and (B) effect of DODAB concentration on the T_s_-, T_m_-, and T_p_-enthalpies.

**Figure 8:**
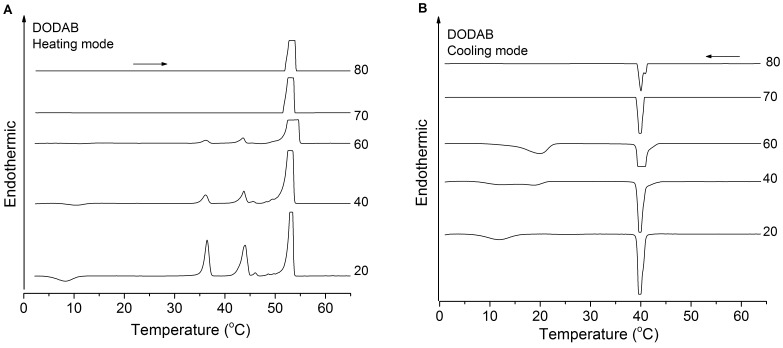
Effect of DODAB concentration on the thermogam profile. Effect of DODAB concentration on the heating (A) and cooling (B) thermograms. Pre-scan time 15 min for all scans. Numbers 20 to 80 besides the curves refer to DODAB concentration (mM). The horizontal arrows indicate heating or cooling scans.

The enthalpies of these transitions are: ΔH_s_ = 39 kJ/mol and ΔH_m_ = 46 kJ/mol, ΔH’_s_ = 31 kJ/mol and ΔH’_m_ = 51 kJ/mol. One should here note that ΔH’_m_>ΔH_m_, which is due to a contribution to ΔH’_m_ from the T’_p_-transition, indicating that the 40°C-exotherm is due to both the T’_m_ and T’_p_-exotherms.

### Co-existence of Unilamellar Vesicles (ULVs) and Multilamellar Structures (MLSs)

Between 1 and 65 mM, the intensity of the T_p_-endotherm increases, while those for the T_s_- and T_m_-endotherms decrease, with increasing DODAB concentration and equilibration time. Within this concentration range, the fraction of ULVs thus decreases while that of MLSs increases as DODAB concentration is raised. The effect of equilibration time furthermore illustrates the slow kinetics of the LC-to-SG transition, as reported also previously [10], [11], [16]. Overall, the height of the T_p_-endotherm is larger for equilibrated than for fresh samples, while the opposite is true for the T_s_- and T_m_-endotherms, indicating the slow formation of MLSs. A sequence of heating/cooling cycles, however, tends to inhibit the T_p_-endotherm and to increase the relative enthapies of the T_s_- and T_m_-endotherms, as shown in Figures 3 and 4.

**Figure 9:**
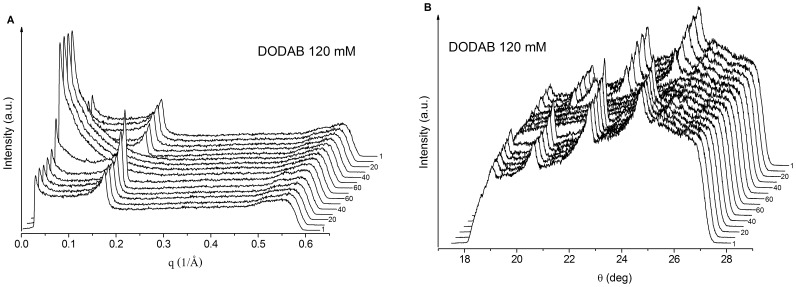
SAXS (A) and WAXS (B) curves for DODAB 120 mM in water, obtained at 1, 10, 20, 30, 40, 50, 60, 70, 60, 50, 40, 30, 20, 10, and 1°C, respectively, as indicated by the numbers besides the curves. Offset between two successive traces is 0.01 and 0.18°C for (a) and (b), respectively.

The thermograms for a fresh DODAB dispersion at 5 mM (not shown) resemble those for 1 mM (Figure 2B), indicating that the dispersion contains mainly ULVs. After equilibrating for 45 days at 25°C, however, the T_p_-endotherm at 53°C is clearly discernible (Figure 5A), indicating the presence of MLSs which transform into ULVs on heating past T_p_ owing to the SG-to-LC transition of the lipids in these structures. As discussed further below, this is indeed compatible with the SAXS data.

**Figure 10:**
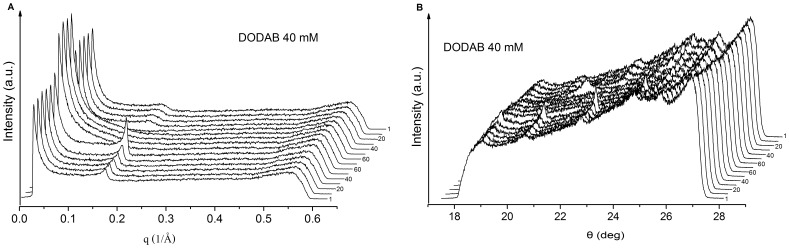
SAXS (A) and WAXS (B) curves for DODAB 40 mM in water, obtained at 1, 10, 20, 30, 40, 50, 60, 70, 60, 50, 40, 30, 20, 10, and 1°C, respectively, as indicated by the numbers besides the curves. Offset between two successive traces is 0.01 and 0.13°C for (a) and (b), respectively.

Further increase in DODAB concentration results in an increase in the T_p_-endotherm and a corresponding decrease in the T_s_- and T_m_-endotherms. At 10 mM, for example, the height of the T_p_-endotherm of a fresh dispersion is comparable to those for the T_s_- or T_m_-endotherms (Figure 5B). In subsequent scans, the enthalpy of the T_p_-endotherm decreased, while those at T_s_ and T_m_ increased (Figure 5B), indicating that the amount of MLSs was reduced after thermal cycling, again due to the slow LC-to-SG transition [11], [16]. These data clearly indicate a higher fraction of MLSs at 10 than at 5 mM, and also in well equilibrated samples.

The effect of pre-scan time is shown in Figure 3 for a fresh 10 mM DODAB dispersion. As seen, there is a pronounced change in enthalpy after the first thermal cycle. Subsequently, the T_p_-enthalpy decreases smoothly to a plateau after 5 h, while the T_s_- and T_m_-enthalpies increase quickly to a plateau. These data illustrate that an equilibration time larger than 16 h is necessary to complete the LC-to-SG transition.

Figure 6 shows two pictures of the same 10 mM DODAB dispersion taken at 25°C, one freshly prepared (top left) and the other equilibrated for three days at 25°C (top right), along with their characteristic heating and cooling thermograms. Together with the DSC and X-ray data, the transition from a clear to a milky system with equilibration indicates that the system is initially dominated by ULVs, which revert to MLSs after equilibration.

Figure 7A shows the effect of the number of thermal cycles on the enthalpy of the T_s_-, T_m_-, and T_p_-endotherms for DODAB 10 mM. On increasing the number of cycles, the T_p_-enthalpy decreases, while the T_s_- and T_m_-enthalpies increase, indicating that freshly cooled dispersions are rich in ULVs, while MLSs dominate in equilibrated dispersions, as shown also in Figure 6. The concentration obviously plays an important role for the structural organization of DODAB aggregates in this concentration range. Overall, as DODAB concentration increases, the T_p_-enthalpy increases, while the T_s_- and T_m_-enthalpies decrease to disappear around 65 mM (Figure 7B). (The decrease in T_p_-enthalpy above 30 mM DODAB is an artifact due to the high intensity of the T_p_-peak which appears truncated in the thermograms (Figure 8)).

The T_m_- and T_s_-endotherms at 45 and 36°C, respectively, vanish when DODAB concentration approaches 65 mM (Figure 8A), indicating that with increasing concentration, ULVs are progressively replaced by MLSs. In the cooling mode, both the T’_s_ and T’_m_-exotherms can be distinguished in the mixed region (Figure 8B). At 20 mM DODAB, the T_s_-, T_m_-, T_p_-, T’_s_- and T’_m_-enthalpies are 19, 23, 43, 13 and 35 kJ/mol, respectively. Again, the T’_m_-enthalpy is larger than the T_m_-enthalpy, indicating that in the T’_m_-exotherm there is also a contribution from the reverse T’_p_-transition.

### Multilamellar Structures (MLSs)

Beyond 65 mM, the dispersions are dominated by MLSs in the SG or LC state, respectively, below or above T_p_ in the heating, or below and above T’_p_ in the cooling process. Upon heating from 1 to 65°C, the thermograms of the MLS dispersions display a single T_p_-endotherm at 53°C, characteristic of the SG-to-LC transition [11], in a similar way to samples from the mixed (MLV + MLS) region. Upon cooling, the thermograms display a single T’_p_-exotherm at 40°C, due to the LC-to-SG reverse transition. The SG-LC transitions for the MLSs thus present a thermal hysteresis ΔT_p_ = 13°C.

The lower boundary (65 mM) of the single MLS region (Figure 1) is lower than previously reported in literature [11], [13], probably because the samples were submitted to equilibration times sufficiently long for the MLS LC-to-SG transition to be complete before starting the heating scans.

### X-ray Scattering

Samples rich in MLSs were also investigated by SAXS and WAXS as a function of temperature (Figure 9). On increasing the temperature from 1 to 50°C, the SAXS curves for 120 mM DODAB (Figure 9A) show a well-defined Bragg peak with peak maximum at 0.172 Å^−1^ independently of temperature, which gives a lattice distance *d* = 36.7 Å, in good agreement with previously reported data [11], [16]. As the temperature approaches T_p_≈53°C, the Bragg peak becomes sharper and more intense, indicating structural growth. No peak was obtained at 60 and 70°C, indicating that above T_p_≈53°C, the multilamellar structures in the LC state transform into unilamellar bilayers (probably ULVs).

On cooling, the Bragg peak starts appearing at the same position at 40°C, in good accordance with the DSC data, presenting a single exotherm at T’_p_ = 40°C (Figure 8). The intensity of the Bragg peaks obtained in the cooling is lower than those in the heating process. This also might be related to the slow kinetics of subgel formation in the cooling process, as indicated by DSC (Figure 8) and also reported elsewhere [11], [16]. Similar behavior was obtained for the SAXS and WAXS curves for 40 mM DODAB dispersion, within the ULV + MLS region, except for the occurrence of lower intensity peaks (Figure 10), indicating that the MLSs are similar in both the single MLS and mixed ULV/MLS dispersions.

Using synchrotron X-ray radiation, we previously observed a Bragg peak at a DODAB concentration of 5.0 mM, but not at 1.0 mM [14], indicating that the onset of MLS formation lies between 1 and 5 mM, in good agreement with the present DSC results.

The extended chain length of DODAB can be estimated from *l*_max_ = 1.5+1.265*n*_c_ [17], where *n*_c_ is the number of hydrocarbons per lipid chain. This gives *l*_max_ = 24.8 Å, suggesting a bilayer thickness of 50 Å. Since *d* was found to be considerably smaller than this (37 Å), our SAXS data suggest that the DODAB molecules are interdigitated in the MLS bilayers. A similar conclusion was previously reached for dilute (5 mM) DODAB dispersions [14]. Because of this, the “melting” temperature (T_p_) of DODAB in MLSs is likely to be larger than that in ULVs (T_m_), as also reported for 1,2-dihexadecyl-*sn*-glycero-3-phosphatidylcholine bilayers [18].

Since there is no change in the Bragg peak position of DODAB MLSs up to 50°C, the gel-to-LC (chain “melting”) temperature (T_p_) must be above 52°C. This suggests that T_p_ ≈ 53°C is actually the “melting” temperature of DODAB in MLSs, equivalently to T_m_ ≈ 45°C for ULVs.

The WAXS curves (Figure 9B) also support these findings. Up to 50°C, there are four well-defined peaks characteristic of macroscopically ordered structures, such as MLSs (or coagel), while above 50°C, there is no peak at all, characteristic of isotropic structures, such as ULVs. In the cooling process, a reverse thermal behavior was observed, in good accord with that obtained by SAXS (Figure 9A).

In Figure 10A and 10B we see similar SAXS and WAXS curve profiles for DODAB at 40 mM, in the ULV/MLS mixed dispersions, indicating identical structures to the single MLS dispersion.

### Conclusions

Based on DSC and SAXS/WAXS, preferred structures in DODAB dispersions were found to depend on concentration, temperature, and sample history. Up to 1 mM, ULVs dominate, displaying two heating endotherms at T_s_≈36°C and T_m_≈45°C, and their cooling reverses at T’_s_≈14°C and T’_m_≈40°C, respectively. Above 65 mM, the dispersion is dominated by MLSs and ULVs, respectively, below and above T_p_ = 53°C in the heating, or below and above T’_p_≈40°C in the cooling process. The subgel-liquid crystalline transitions in MLSs thus display hysteresis ΔT_p_≈13°C. Interestingly, T_p_>T_m_ while T’_p_ = T’_m_. The larger T_p_ value was related to an interdigitated conformation of DODAB chains in the MLS bilayers. ΔT_p_>ΔT’_m_ indicates the chain “freezing” (reverse to “melting”) is slower for MLSs than for ULVs, probably because of the chain interdigitation. In the intermediate 1–65 mM, MLSs and ULVs co-exist. It was also shown that sample thermal history plays important role for the structures formed in this system, demonstrated to be due the slow LC-to-SG transition. Finally, it was shown that T_p_≈53°C is actually the “melting” temperature of DODAB in MLSs, equivalently to T_m_≈45°C for ULVs.

## Supporting Information

Table S1 **Summary of DODAB aggregate structures at different temperature and DODAB concentration.** Here, CS refers to samples cooled from T_f_ = 65°C, WS to samples warmed from T_i_ = 1°C, LC to Liquid-crystalline, ULV to unilamellar vesicles, and MLS to multilemellar structures.
(DOC)
