# Supplementary material for: Thermal and Structural Behavior of Dioctadecyldimethylammonium Bromide Dispersions Studied by Differential Scanning Calorimetry and X-Ray Scattering
Source: PLoS One. 2012 Sep 6;7(9):e44702. doi: 10.1371/journal.pone.0044702 (PMC3435325; doi:10.1371/journal.pone.0044702)
Supplement: Table S1 — Summary of DODAB aggregate structures at different temperature and DODAB concentration. Here, CS refers to samples cooled from Tf = 65°C, WS to samples warmed from Ti = 1°C, LC to Liquid-crystalline, ULV to unilamellar vesicles, and MLS to multilemellar structures. (DOC) [file pone.0044702.s001.doc]

**Table S1**

| **Temp (^o^C)** | **< 1 mM** | **1–65 mM** | **> 65 mM** |
| --- | --- | --- | --- |
| **> 65 ^o^C** | ULV in the LC state (not investigated) | ULV in the LC state  (not investigated) | ULV in the LC state  (investigated up to 70 ^o^C by SAXS) |
| T_f_ ≈ 65 ^o^C: Temperature of sample preparation | | | |
| **LC state** | ULV in the LC state | ULV in the LC state | ULV in the LC state |
| T_p_ ≈ 53 ^o^C: MLS SG-to-LC transition temperature | | | |
| **LC state** | ULV in the LC state | ULV in the LC state  and  MLS in the SG (WS) or ULV in the LC state (CS) | MLS in the SG (WS) or ULV in the LC state (CS) |
| T_m_ ≈ 45 ^o^C: ULV Gel-to-LC transition temperature | | | |
| **Gel or LC state** | ULV in the gel (WS) or LC (CS) state | ULV in the gel (WS) or ULV state (CS)  and  MLS in the SG (WS) or ULVstate (CS) | MLS in the SG state (WS) or ULV in the LC state (CS) |
| T’_m_ = T’_p_ ≈ 40 ^o^C: MLV and MLS LC-to-gel transition temperature | | | |
| **Gel state** | ULV in the gel state | ULV in the gel state  and  MLS in the SG state | MLS in the SG state |
| T_s_ ≈ 36 ^o^C: SG-to-gel transition temperature | | | |
| **Gel or SG state** | ULV in the SG (WS) or gel (CS) state | ULV in the SG (WS) or gel (CS) state  and  MLS in the SG state | MLS in the SG state |
| T’_s_ ≈ 13 ^o^C: Gel-to-SG transition temperature | | | |
| **SG state** | ULV in the SG phase | ULV in the SG state  and  MLS in the SG state | MLS in the SG state |
| T_i_ = 1 ^o^C: Temperature of starting DSC heating scan | | | |
| **< 1 ^o^C** | Not investigated | Not investigated | Not investigated |
